# Association between lactate-to-albumin ratio and 30-day all-cause mortality in patients with acute pancreatitis-associated acute kidney injury

**DOI:** 10.1038/s41598-026-42882-5

**Published:** 2026-03-11

**Authors:** Mingjun Wei, Yue Zhong, Xiao Lin, Beilei Zhang

**Affiliations:** 1 Department of Critical Care Medicine, The First Affiliated Hospital of Fujian Medical University, NO. 20, Chazhong Road, Taijiang District, Fuzhou, 350005 Fujian Province People’s Republic of China; 2 National Regional Medical Center, Binhai Campus of the First Affiliated Hospital, Fujian Medical University, Fuzhou, 350212 People’s Republic of China

**Keywords:** Lactate/albumin ratio, Acute pancreatitis, Acute kidney injury, All-cause mortality, 30-days, Prognosis

## Abstract

In recent years, the lactate-to-albumin ratio (LAR) has emerged as a novel prognostic marker in various critical illnesses, including sepsis, heart failure, and acute respiratory failure. However, its predictive value in patients with acute pancreatitis (AP)—particularly those who develop acute pancreatitis–associated acute kidney injury (AP-AKI)—has not been fully evaluated. Given the high short-term all-cause mortality observed in AP-AKI patients, this study aims to examine the association between admission LAR levels and 30-day all-cause mortality in this population, with the goal of providing new evidence for clinical risk stratification and early intervention. We performed a retrospective cohort study of 877 critically ill patients with AP-AKI using the MIMIC-IV/III database, and used the eICU-CRD database and a cohort of AP-AKI patients admitted to the Department of Critical Care Medicine, First Affiliated Hospital of Fujian Medical University (*FAHFMU*) for external validation. Patients were stratified into four groups according to LAR quartiles. Confounding was assessed via linear or logistic regression with stepwise covariate removal, and univariable and multivariable Cox models were used to identify predictors of mortality. ROC analysis (AUC) evaluated LAR and other markers, establishing an optimal cut-off for Kaplan–Meier survival curves (log-rank test). Restricted cubic spline (RCS) functions were used to explore the potential nonlinear relationship between LAR and 30-day mortality. Subgroup analyses were conducted across age, sex, comorbidities, and treatment categories. The study found that higher LAR levels were significantly associated with increased 30-day mortality in AP-AKI patients. After adjusting for potential confounders, LAR remained an independent predictor of mortality (HR 1.172, 95% CI 1.054–1.303, *P* < 0.05). ROC analysis showed an AUC of 0.695 for LAR, surpassing that of lactate alone (0.683) or albumin (0.589), and comparable to the SOFA score (0.684). Compared with the low LAR group, the high LAR group had a significantly higher mortality rate (*P* < 0.001), and a nonlinear association between LAR and 30-day mortality was observed. LAR is an independent predictor of 30-day all-cause mortality in AP-AKI. Given its simplicity and accessibility yet modest discrimination, LAR may serve as a useful adjunct for early risk assessment, particularly when combined with established scores or used serially.

**Supplementary Information:**

The online version contains supplementary material available at 10.1038/s41598-026-42882-5.

## Introduction

Acute pancreatitis (AP) is a common gastrointestinal disorder, with an annual incidence ranging from approximately 13 to 74 cases per 100,000 individuals. While most cases are mild and self-limiting, about 20% of patients progress to severe acute pancreatitis (SAP), which is associated with high mortality rates^1^. Acute kidney injury (AKI) is a frequent complication of SAP, occurring in up to 15% of cases and contributing to mortality rates as high as 50%^2^. The development of AKI in SAP patients is linked to factors such as hypovolemia, systemic inflammatory response syndrome (SIRS), and the direct nephrotoxic effects of inflammatory cytokines^3^. Therefore, early identification of high-risk patients is crucial for improving outcomes.

Ranson, APACHE II, and SOFA remain commonly used tools for assessing the severity of AP^4^.In parallel, there is growing interest in simple and readily available biomarkers that can support early risk flagging at the bedside. Within this context, the lactate-to-albumin ratio (LAR) has emerged as a pragmatic candidate given its routine availability and biological plausibility.

Lactate is a marker of tissue hypoperfusion and cellular hypoxia, and elevated levels have been associated with increased mortality in critically ill patients^5^. Albumin, a negative acute-phase reactant, reflects nutritional status and inflammation levels; hypoalbuminemia has been linked to poor outcomes in both AP and AKI^6,7^. The LAR combines the advantages of these two indicators and has demonstrated superior predictive ability over lactate or albumin alone in sepsis-related AKI^8,9^. Furthermore, studies have shown that AP and sepsis share similarities in systemic inflammation and organ dysfunction, suggesting that LAR may also have prognostic value in AP patients with AKI^10^.

Consequently, LAR may be a promising composite biomarker for evaluating the severity and predicting the prognosis of AP-AKI. Nonetheless, existing studies exploring the link between LAR and clinical outcomes in AP-AKI patients are still scarce. To address this research gap, the present study utilizes data from the MIMIC-IV (version 3.1) and MIMIC-III (version 1.4) databases for further analysis, supplemented by the e-ICU database and a cohort of AP-AKI patients admitted between 2022 and 2025 to the Department of Critical Care Medicine at the First Affiliated Hospital of Fujian Medical University. The primary objective is to investigate the relationship between LAR and patient outcomes, thereby providing new insights into the management of AP-AKI patients and offering a simple and effective biomarker for early clinical risk assessment.

## Materials and methods

### Data sources

For the purpose of this retrospective analysis, data were sourced from the publicly available Medical Information Mart for Intensive Care databases—MIMIC-IV (version 3.1) and MIMIC-III (version 1.4)—which are specifically curated to support clinical research^11,12^. Data extraction was performed using Structured Query Language (SQL) implemented within the PostgreSQL environment. For external validation, we used two datasets from a different source. This dataset was derived from the eICU Collaborative Research Database (version 2.0, data collection period: 2014 to 2015), a large multi-center intensive care database developed through a collaboration between Philips Healthcare and the Laboratory for Computational Physiology at the Massachusetts Institute of Technology (MIT). The second dataset consisted of patients diagnosed with acute pancreatitis-associated acute kidney injury (AP-AKI) who were admitted between 2022 and 2025 to the Department of Critical Care Medicine at the First Affiliated Hospital of Fujian Medical University. Access to the MIMIC-IV/III and eICU-CRD databases was granted following the completion of the mandatory training by the principal investigator through the National Institutes of Health platform. This included certification in the courses “Study Data or Specimens Only” and “Conflict of Interest” (certification IDs: 66,955,426 and 66,955,425).

### Study population

**Fig. 1:**
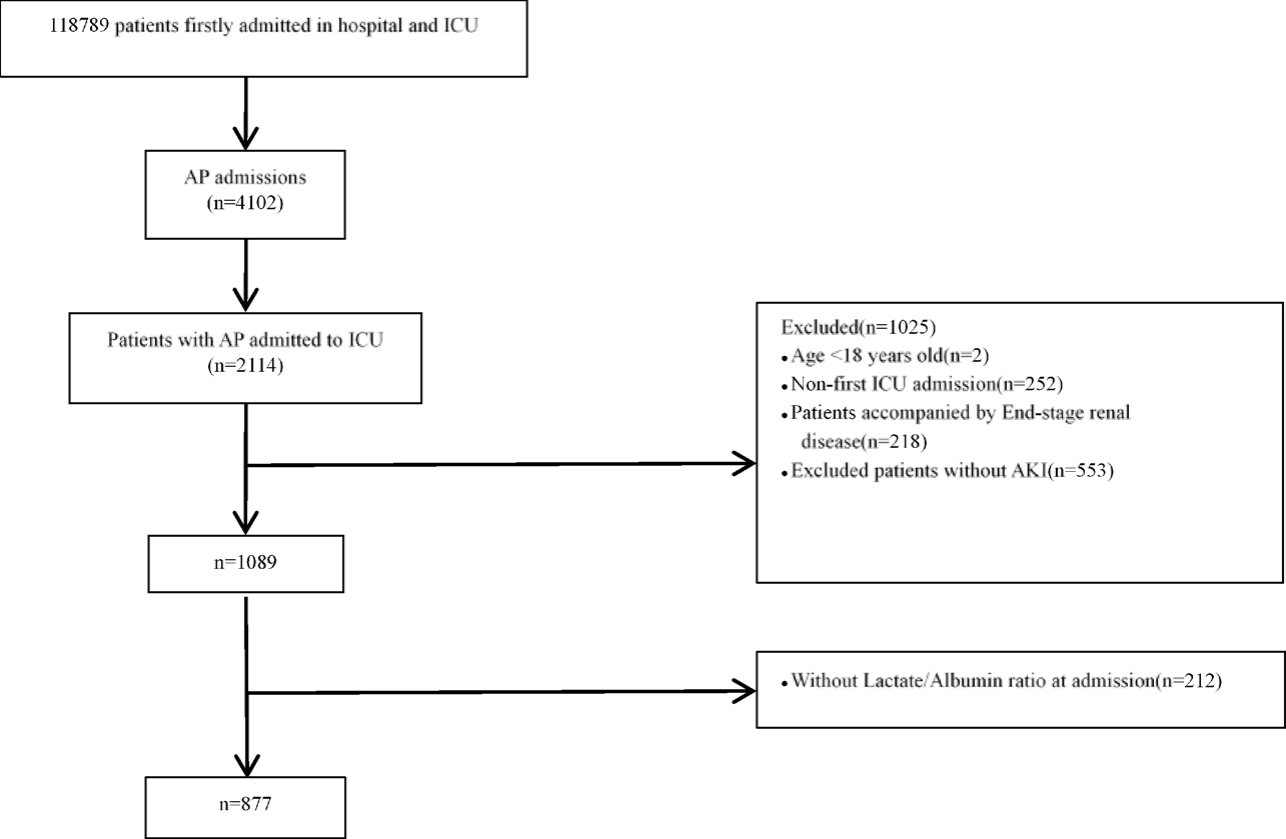
Schematic diagram of study sample selection steps. AP, Acute Pancreatitis; ICU, intensive care unit; AKI, Acute Kidney Injury; LAR, Lactate/Albumin Ratio.

According to the Massachusetts Institute of Technology Institutional Review Board, the MIMIC-IV, MIMIC-III, and eICU-CRD databases are exempt from additional ethical review due to the de-identification of patient information, which ensures data privacy. This study adhered to the ethical principles outlined in the Declaration of Helsinki. Among the 756,394 total hospitalizations documented in the MIMIC-IV database, 118,789 patients were identified with initial hospital and ICU admissions. Patient admission data for acute pancreatitis (AP) were identified using the International Classification of Diseases, 10th Revision (ICD-10) codes K85–K85.92, and 9th Revision (ICD-9) code 577.0. A total of 4,102 individuals were initially included, of whom 2,114 had ICU admissions. After applying exclusion criteria, the following patients were removed from the cohort: (1) individuals under 18 years of age at the time of initial admission; (2) patients with multiple admissions for AP, retaining only data from the first hospitalization; (3) individuals diagnosed with end-stage renal disease; (4) those who did not develop AKI during their hospital stay; and (5) patients lacking blood lactate and serum albumin measurements within the first 24 h of admission. Following this screening process, 877 patients met the inclusion criteria and were incorporated into the final analysis (Fig. 1). We included 127 cases from the eICU-CRD and 149 from the ICU of the First Affiliated Hospital of Fujian Medical University.

### Data extraction

Medical data were extracted from the MIMIC-IV, MIMIC-III and eICU-CRD databases using DecisionLink Full software (version 1.1.5.9), while data from the First Affiliated Hospital of Fujian Medical University were obtained from the hospital’s medical record system. Collected clinical variables encompassed demographic information (age, sex), clinical outcomes (mortality), and physiological/laboratory parameters such as heart rate, hematocrit, hemoglobin, and international normalized ratio (INR). Documented comorbidities included hypertension, myocardial infarction, chronic pulmonary disease, malignancy, and liver cirrhosis. Therapeutic interventions were recorded, including administration of anti-infective agents, vasopressors (e.g., norepinephrine and dopamine), and loop diuretics. Disease diagnoses were coded based on the International Classification of Diseases, 9th and 10th Revision, Clinical Modification (ICD-9-CM and ICD-10-CM).

### Exposure

The LAR used as a risk indicator, was derived by dividing serum lactate concentration by serum albumin concentration. LAR was calculated using the first lactate (mmol/L) and albumin (g/dL) values obtained within the first 24 h after ICU admission; SOFA was computed from the same 24-h window to ensure temporal alignment. To facilitate analysis, LAR values were divided into quartiles based on their distribution: values less than 0.44 were assigned to the first quartile; those ranging from 0.44 to 0.69 comprised the second quartile; the third quartile included values from 0.69 to 1.22; and values equal to or exceeding 1.22 were placed in the fourth quartile.

### Study endpoints

The primary outcomes were in-hospital and 30-day mortality. Acute kidney injury (AKI) was identified according to KDIGO criteria using both serum creatinine and urine output^13^. We also documented AKI onset timing as either present on ICU admission or developed during hospitalization. No stage-specific subgroup analyses were prespecified.

### Statistical analysis

In the descriptive analysis, participants were divided into four groups based on the quartile distribution of their LAR values. Continuous variables were summarized using either the mean ± standard deviation or the median with interquartile range (IQR), and intergroup differences were evaluated using one-way ANOVA or the Kruskal–Wallis test, depending on data distribution. Categorical variables were reported as frequencies and percentages, with comparisons made using the Chi-square test or Fisher’s exact test, as appropriate. To account for potential confounding factors, covariates were included in baseline models utilizing either linear or binary logistic regression. These covariates were subsequently removed in a stepwise manner from the full model to assess their influence on regression coefficients. To identify potential risk factors for in-hospital mortality, univariable Cox proportional hazards regression was initially performed. Variables with a *p*-value < 0.05 were subsequently entered into a multivariable Cox regression model to determine independent predictors. Receiver Operating Characteristic (ROC) curve analysis was conducted to evaluate the predictive performance of lactate, albumin, the lactate-to-albumin ratio (LAR), and the Sequential Organ Failure Assessment (SOFA) score for 30-day mortality, including the calculation of sensitivity, specificity, and the area under the curve (AUC). Discrimination for in-hospital mortality was evaluated similarly using ROC curves. Based on the median LAR value, patients were stratified into high and low LAR groups. Kaplan–Meier survival curves were generated for these groups, and differences were assessed using the log-rank test. To explore potential nonlinear associations between LAR and 30-day mortality, restricted cubic spline (RCS) modeling was applied. Additionally, subgroup analyses were performed to evaluate the effect of LAR across various clinical subgroups, including age, sex, hypertension, type 2 diabetes mellitus (T2DM), cancer, ischemic heart disease (IHD), and use of diuretics or vasopressors. All statistical analyses were executed using R software (version 4.2.2, The R Foundation, http://www.R-project.org) and DecisionLink Full (version 1.1.5.9). A two-sided *p*-value of less than 0.05 was considered statistically significant.

## Results

### Baseline demographic and clinical characteristics

In this study, we analyzed the baseline characteristics of 877 patients divided into four groups based on LAR quartiles, with cutoff points of < 0.44, 0.44–0.69, 0.69–1.22, and > 1.22 (Table 1). The median age of the cohort was 57 years (IQR 45–70), and the gender distribution was 42.87% female and 57.13% male, with no significant differences observed between LAR groups (*p* = 0.658). In the eICU-CRD validation cohort, the median age was 55 years, males accounted for 61.4%, and the 30-day mortality rate was 18.9%. In the validation cohort from the First Affiliated Hospital of Fujian Medical University, the median age was 64 years, 68.5% of the patients were male, and the 30-day mortality rate was 19.5%.

**Table 1:** Baseline characteristics among LAR quartiles.

Variable	Total(n = 877)	LAR quartiles				*p*
		< 0.44(n = 221)	≥ 0.44, and < 0.69(n = 218)	≥ 0.69, and < 1.22(n = 219)	≥ 1.22(n = 219)	
LAR, median (IQR)	0.69 (0.44–1.22)	0.35 (0.29–0.40)	0.54 (0.50–0.59)	0.90 (0.78–1.04)	2.00 (1.56–2.88)	< 0.001
Age (yr)	57.00 (45.00–70.00)	54.00 (42.00–67.00)	60.00 (47.00–71.00)	58.00 (46.00–71.00)	57.00 (46.00–68.00)	0.032
Weight (kg)	85.20 (72.10–100.97)	85.00 (71.50–101.20)	86.40 (73.57–99.80)	84.20 (71.00–101.00)	86.00 (72.60–101.13)	0.762
Sofa score, median (p25–p75)	7.00 (4.00–10.00)	5.00 (3.00–8.00)	5.00 (3.00–8.00)	7.00 (4.00–10.00)	10.00 (6.00–13.00)	< 0.001
Saps II score, median (p25–p75)	39.00 (30.00–52.00)	33.00 (25.00–43.00)	35.00 (27.00–45.00)	39.00 (31.00–51.00)	52.00 (39.00–64.00)	< 0.001
Oasis score, median (p25–p75)	36.00 (30.00–43.00)	33.00 (28.00–39.00)	34.00 (29.00–40.00)	36.00 (30.00–42.00)	42.00 (33.00–49.00)	< 0.001
Hct (%)	33.70 (29.20–39.20)	33.40 (29.10–38.30)	33.95 (29.80–38.80)	33.60 (29.00–39.90)	34.00 (28.80–40.00)	0.818
Hb (g/dL)	11.20 (9.60–13.20)	11.10 (9.60–12.80)	11.30 (9.90–13.20)	11.20 (9.60–13.30)	11.10 (9.30–13.20)	0.829
Plt (× 10^9^ /L)	188.00 (126.00–272.00)	188.00 (138.00–309.00)	206.50 (146.00–279.00)	198.00 (132.00–270.00)	159.00 (105.00–247.00)	< 0.001
Alb (g/dL)	2.80 (2.40–3.20)	3.00 (2.60–3.50)	2.95 (2.50–3.40)	2.70 (2.20–3.10)	2.50 (2.10–2.90)	< 0.001
Lac (mmol/L)	1.90 (1.30–3.40)	1.00 (0.90–1.20)	1.60 (1.30–1.90)	2.30 (1.90–2.90)	4.90 (3.90–7.20)	< 0.001
INR	1.30 (1.20–1.70)	1.30 (1.10–1.40)	1.30 (1.20–1.50)	1.40 (1.20–1.60)	1.60 (1.30–2.20)	< 0.001
Gender, n (p%)						0.658
Female	376.00 (42.87%)	90.00 (40.72%)	90.00 (41.28%)	101.00 (46.12%)	95.00 (43.38%)	
Male	501.00 (57.13%)	131.00 (59.28%)	128.00 (58.72%)	118.00 (53.88%)	124.00 (56.62%)	
Hypertension, n (p%)						0.424
No	461.00 (52.57%)	106.00 (47.96%)	121.00 (55.50%)	116.00 (52.97%)	118.00 (53.88%)	
Yes	416.00 (47.43%)	115.00 (52.04%)	97.00 (44.50%)	103.00 (47.03%)	101.00 (46.12%)	
Cirrhosis, n (p%)						0.001
No	770.00 (87.80%)	204.00 (92.31%)	198.00 (90.83%)	191.00 (87.21%)	177.00 (80.82%)	
Yes	107.00 (12.20%)	17.00 (7.69%)	20.00 (9.17%)	28.00 (12.79%)	42.00 (19.18%)	
Stroke, n (p%)						0.315
No	834.00 (95.10%)	209.00 (94.57%)	211.00 (96.79%)	210.00 (95.89%)	204.00 (93.15%)	
Yes	43.00 (4.90%)	12.00 (5.43%)	7.00 (3.21%)	9.00 (4.11%)	15.00 (6.85%)	
Cancer, n (p%)						0.946
No	815.00 (92.93%)	207.00 (93.67%)	203.00 (93.12%)	202.00 (92.24%)	203.00 (92.69%)	
Yes	62.00 (7.07%)	14.00 (6.33%)	15.00 (6.88%)	17.00 (7.76%)	16.00 (7.31%)	
T2DM, n (p%)						0.902
No	671.00 (76.51%)	166.00 (75.11%)	169.00 (77.52%)	166.00 (75.80%)	170.00 (77.63%)	
Yes	206.00 (23.49%)	55.00 (24.89%)	49.00 (22.48%)	53.00 (24.20%)	49.00 (22.37%)	
IHD, n (p%)						0.801
No	789.00 (89.97%)	202.00 (91.40%)	193.00 (88.53%)	197.00 (89.95%)	197.00 (89.95%)	
Yes	88.00 (10.03%)	19.00 (8.60%)	25.00 (11.47%)	22.00 (10.05%)	22.00 (10.05%)	
COPD, n (p%)						0.928
No	818.00 (93.27%)	205.00 (92.76%)	204.00 (93.58%)	203.00 (92.69%)	206.00 (94.06%)	
Yes	59.00 (6.73%)	16.00 (7.24%)	14.00 (6.42%)	16.00 (7.31%)	13.00 (5.94%)	
Diuretic, n (p%)						0.002
No	440.00 (50.17%)	92.00 (41.63%)	101.00 (46.33%)	121.00 (55.25%)	126.00 (57.53%)	
Yes	437.00 (49.83%)	129.00 (58.37%)	117.00 (53.67%)	98.00 (44.75%)	93.00 (42.47%)	
Pressors, n (p%)						< 0.001
No	164.00 (18.70%)	55.00 (24.89%)	47.00 (21.56%)	42.00 (19.18%)	20.00 (9.13%)	
Yes	713.00 (81.30%)	166.00 (75.11%)	171.00 (78.44%)	177.00 (80.82%)	199.00 (90.87%)	
Antibiotic, n (p%)						0.028
No	27.00 (3.08%)	13.00 (5.88%)	4.00 (1.83%)	7.00 (3.20%)	3.00 (1.37%)	
Yes	850.00 (96.92%)	208.00 (94.12%)	214.00 (98.17%)	212.00 (96.80%)	216.00 (98.63%)	
Ventilation, n (p%)						0.166
No	198.00 (22.58%)	62.00 (28.05%)	45.00 (20.64%)	45.00 (20.55%)	46.00 (21.00%)	
Yes	679.00 (77.42%)	159.00 (71.95%)	173.00 (79.36%)	174.00 (79.45%)	173.00 (79.00%)	
In-hospital mortality, n (p%)						< 0.001
No	529.00 (60.32%)	162.00 (73.30%)	137.00 (62.84%)	125.00 (57.08%)	105.00 (47.95%)	
Yes	348.00 (39.68%)	59.00 (26.70%)	81.00 (37.16%)	94.00 (42.92%)	114.00 (52.05%)	
30-day mortality, n (p%)						< 0.001
No	781.00 (89.05%)	213.00 (96.38%)	206.00 (94.50%)	188.00 (85.84%)	174.00 (79.45%)	
Yes	96.00 (10.95%)	8.00 (3.62%)	12.00 (5.50%)	31.00 (14.16%)	45.00 (20.55%)	

*LAR* Lactate-to-Albumin Ratio; *IQR* Interquartile range; *SOFA* Sequential organ failure assessment; *SAPS II* Simplified Acute Physiology Score II; *OASIS* Oxford acute severity of illness score; *Hct* Hematocrit; *Hb* Hemoglobin; *Plt* Platelet count; *Alb* Albumin; *Lac* Lactate; *INR* International normalized ratio; *T2DM* Type 2 diabetes mellitus; *IHD* Ischemic heart disease; *COPD* Chronic obstructive pulmonary disease.

Severity scores, including SAPS II, SOFA, and OASIS, showed a significant upward trend across increasing LAR quartiles, reaching the highest values in the group with LAR > 1.22 (*p* < 0.001). Regarding treatments and comorbidities, patients with elevated LAR were more frequently treated with vasopressors (*p* < 0.001), loop diuretics (*p* = 0.002), and antibiotics (*p* < 0.028). Additionally, the prevalence of cirrhosis increased progressively with higher LAR levels (*p* < 0.001). Moreover, patients with higher LAR levels also have higher INR and platelet levels (*p* < 0.001). In the validation cohort (eICU-CRD), patients were grouped according to 30-day mortality status. Significant differences were observed in LAR, sex, SOFA score, SAPS II score, platelet count (Plt), lactate (Lac), international normalized ratio (INR), hypertension, and mechanical ventilation between survivors and non-survivors (Table S1). In the validation cohort (*FAHFMU*), Patients were stratified by 30-day mortality status, and significant differences were observed between survivors and non-survivors in terms of LAR, platelet count (Plt), albumin (Alb), lactate (Lac), international normalized ratio (INR), hypertension, use of antibiotics, vasopressors, and mechanical ventilation (Table S2).

### LAR is an independent predictor of 30-day all-cause mortality after ICU admission

Covariates showing significant differences in Table 2 (*P* < 0.05) were included in the univariable Cox regression analysis. The findings revealed that the unadjusted lactate-to-albumin ratio (LAR) was significantly correlated with 30-day all-cause mortality following hospital admission (HR: 1.418; 95% CI 1.307–1.538; *P* < 0.001). Variables with *P* < 0.05 in Table 3, along with potential risk factors, were subsequently incorporated into Lasso regression to refine variable selection. Features with a lambda coefficient of zero were excluded, and the remaining variables were entered into multivariable Cox regression models. Table 4 summarizes the adjusted associations between LAR and 30-day in-hospital mortality in acute pancreatitis (AP) patients. In Model I, after adjusting for age, SOFA score, OASIS score, cirrhosis, cancer, and ischemic heart disease (IHD), LAR remained significantly associated with mortality (HR: 1.17; 95% CI 1.049–1.305; *P* = 0.005). Further adjustment for hemoglobin (Hb), platelets (Plt), vasopressors, and diuretics in Model II confirmed LAR as an independent predictor (HR: 1.172; 95% CI 1.054–1.303; *P* = 0.003). Validation in the eICU-CRD cohort using multivariable Cox regression also demonstrated that LAR independently predicted 30-day mortality (HR: 1.37; 95% CI 1.129–1.678; *P* = 0.002) (Table S2). The ROC analysis yielded an area under the curve (AUC) of 0.756 (95% CI 62.0–89.2%). The same conclusion was reached in the *FAHFMU* validation cohort: LAR independently predicted 30-day mortality (HR: 14.076; 95% CI 1.963–100.938; *P* = 0.009), and the ROC analysis yielded an area under the curve (AUC) of 0.907 (95% CI 84.3–97.1%).

**Table 2:** Baseline characteristics between survivors and non-survivors.

Variable	Total(n = 877)	30-dsurvivors (N = 781)	30-d non-survivors (N = 96)	*p*
Age (yr)	57.00 (45.00–70.00)	56.00 (45.00–68.00)	66.50 (52.00–77.00)	< 0.001
Weight (kg)	85.20 (72.10–100.97)	86.00 (72.57–101.00)	84.40 (70.15–96.75)	0.285
Sofa score, median (p25–p75)	7.00 (4.00–10.00)	6.00 (4.00–9.00)	10.00 (6.50–14.00)	< 0.001
Saps II score, median (p25–p75)	39.00 (30.00–52.00)	38.00 (29.00–49.00)	54.00 (41.50–63.00)	< 0.001
Oasis score, median (p25–p75)	36.00 (30.00–43.00)	35.00 (30.00–42.00)	40.00 (33.50–47.00)	< 0.001
Hct (%)	33.70 (29.20–39.20)	34.10 (29.50–39.30)	30.70 (25.55–37.55)	< 0.001
Hb (g/dL)	11.20 (9.60–13.20)	11.30 (9.90–13.20)	9.85 (8.55–12.25)	< 0.001
Plt (× 10^9^ /L)	188.00 (126.00–272.00)	193.00 (131.00–276.00)	161.50 (89.50–260.50)	0.005
Alb (g/dL)	2.80 (2.40–3.20)	2.80 (2.40–3.20)	2.50 (2.00–3.20)	0.002
Lac (mmol/L)	1.90 (1.30–3.40)	1.80 (1.20–3.10)	3.10 (1.80–5.55)	< 0.001
LAR	0.69 (0.44–1.22)	0.63 (0.43–1.13)	1.16 (0.74–2.57)	< 0.001
INR	1.30 (1.20–1.70)	1.30 (1.20–1.60)	1.65 (1.30–2.20)	< 0.001
Gender, n (p%)				0.087
Female	376.00 (42.87%)	327.00 (41.87%)	49.00 (51.04%)	
Male	501.00 (57.13%)	454.00 (58.13%)	47.00 (48.96%)	
Hypertension, n (p%)				0.444
No	461.00 (52.57%)	407.00 (52.11%)	54.00 (56.25%)	
Yes	416.00 (47.43%)	374.00 (47.89%)	42.00 (43.75%)	
Cirrhosis, n (p%)				< 0.001
No	770.00 (87.80%)	707.00 (90.52%)	63.00 (65.63%)	
Yes	107.00 (12.20%)	74.00 (9.48%)	33.00 (34.38%)	
Stroke, n (p%)				0.723
No	834.00 (95.10%)	742.00 (95.01%)	92.00 (95.83%)	
Yes	43.00 (4.90%)	39.00 (4.99%)	4.00 (4.17%)	
Cancer, n (p%)				0.009
No	815.00 (92.93%)	732.00 (93.73%)	83.00 (86.46%)	
Yes	62.00 (7.07%)	49.00 (6.27%)	13.00 (13.54%)	
T2DM, n (p%)				0.157
No	671.00 (76.51%)	592.00 (75.80%)	79.00 (82.29%)	
Yes	206.00 (23.49%)	189.00 (24.20%)	17.00 (17.71%)	
IHD, n (p%)				0.022
No	789.00 (89.97%)	709.00 (90.78%)	80.00 (83.33%)	
Yes	88.00 (10.03%)	72.00 (9.22%)	16.00 (16.67%)	
COPD, n (p%)				0.126
No	818.00 (93.27%)	732.00 (93.73%)	86.00 (89.58%)	
Yes	59.00 (6.73%)	49.00 (6.27%)	10.00 (10.42%)	
Diuretic, n (p%)				< 0.001
No	440.00 (50.17%)	349.00 (44.69%)	91.00 (94.79%)	
Yes	437.00 (49.83%)	432.00 (55.31%)	5.00 (5.21%)	
Pressors, n (p%)				< 0.001
No	164.00 (18.70%)	158.00 (20.23%)	6.00 (6.25%)	
Yes	713.00 (81.30%)	623.00 (79.77%)	90.00 (93.75%)	
Antibiotic, n (p%)				0.221
No	27.00 (3.08%)	26.00 (3.33%)	1.00 (1.04%)	
Yes	850.00 (96.92%)	755.00 (96.67%)	95.00 (98.96%)	
Ventilation, n (p%)				0.001
No	198.00 (22.58%)	189.00 (24.20%)	9.00 (9.38%)	
Yes	679.00 (77.42%)	592.00 (75.80%)	87.00 (90.63%)	

*SOFA* sequential organ failure assessment; *SAPS II* Simplified acute physiology score II; *OASIS* Oxford acute severity of illness score; *Hct* Hematocrit; *Hb* Hemoglobin; *Plt* Platelet count; *Alb* Albumin; *Lac* Lactate; *LAR* Lactate-to-albumin ratio; *INR* International normalized ratio; *T2DM* Type 2 diabetes mellitus; *IHD* Ischemic heart disease; *COPD* Chronic obstructive pulmonary disease.

**Table 3:** Univariable COX analysis of risk factors for death within 30-d in patients.

Variables	Hazard ratio (HR)	95% CI	*P*
LAR	1.418	1.307–1.538	< 0.001
Lac	1.186	1.135–1.24	< 0.001
Alb	0.582	0.421–0.804	0.001
Age	1.026	1.014–1.039	< 0.001
Sofa score	1.169	1.121–1.22	< 0.001
Oasis score	1.045	1.023–1.067	< 0.001
Saps II score	1.044	1.032–1.056	< 0.001
Cancer			
No			
Yes	2.181	1.216–3.914	0.009
Cirrhosis			
No			
Yes	4.261	2.796–6.495	< 0.001
IHD			
No			
Yes	1.869	1.093–3.197	0.022
Diuretic			
No			
Yes	0.05	0.02–0.122	< 0.001
Pressors			
No			
Yes	3.623	1.586–8.279	0.002
Ventilation			
No			
Yes	2.913	1.466–5.785	0.002
INR	1.189	1.06–1.334	0.003
Hb	0.846	0.777–0.92	< 0.001
Hct	0.957	0.931–0.984	0.002
Plt	0.998	0.996–1	0.018

*LAR* Lactate-to-Albumin Ratio; *Lac* Lactate; *Alb* Albumin; *SOFA* Sequential organ failure assessment; *OASIS* Oxford acute severity of illness score; *SAPS II* Simplified acute physiology score II; *IHD* Ischemic heart disease; *INR* International normalized ratio; *Hb* Hemoglobin; *Hct* Hematocrit; *Plt* Platelet count.

**Table 4:** Multivariable COX analysis of risk factors for death in patients within 30-d.

Variables	Hazard ratio (HR)	95% CI	*P*
Model I			
LAR	1.17	1.049–1.305	0.005
Age	1.029	1.012–1.046	0.001
Cirrhosis	3.216	2.003–5.164	< 0.001
Cancer	1.333	0.702–2.531	0.379
IHD	1.48	0.849–2.578	0.166
Saps II score	1.006	0.984–1.028	0.601
Sofa score	1.1	1.019–1.186	0.014
Model II			
LAR	1.172	1.054–1.303	0.003
Age	1.034	1.017–1.051	< 0.001
Cirrhosis	2.557	1.581–4.135	< 0.001
Cancer	1.123	0.602–2.095	0.715
IHD	0.657	0.371–1.163	0.149
Saps II score	1.018	0.998–1.039	0.082
Sofa score	1.004	0.927–1.087	0.927
Diuretic	0.041	0.016–0.102	< 0.001
Pressors	5.144	2.171–12.188	< 0.001
Hb	0.87	0.806–0.94	< 0.001
Plt	0.998	0.996–1	0.074

*LAR* Lactate-to-albumin ratio; *IHD* Ischemic heart disease; *SAPS II* Simplified acute physiology score II; *SOFA* Sequential organ failure assessment; *Hb* Hemoglobin; *Plt* Platelet count.

### ROC curve analysis and Kaplan–Meier curve

**Fig. 2:**
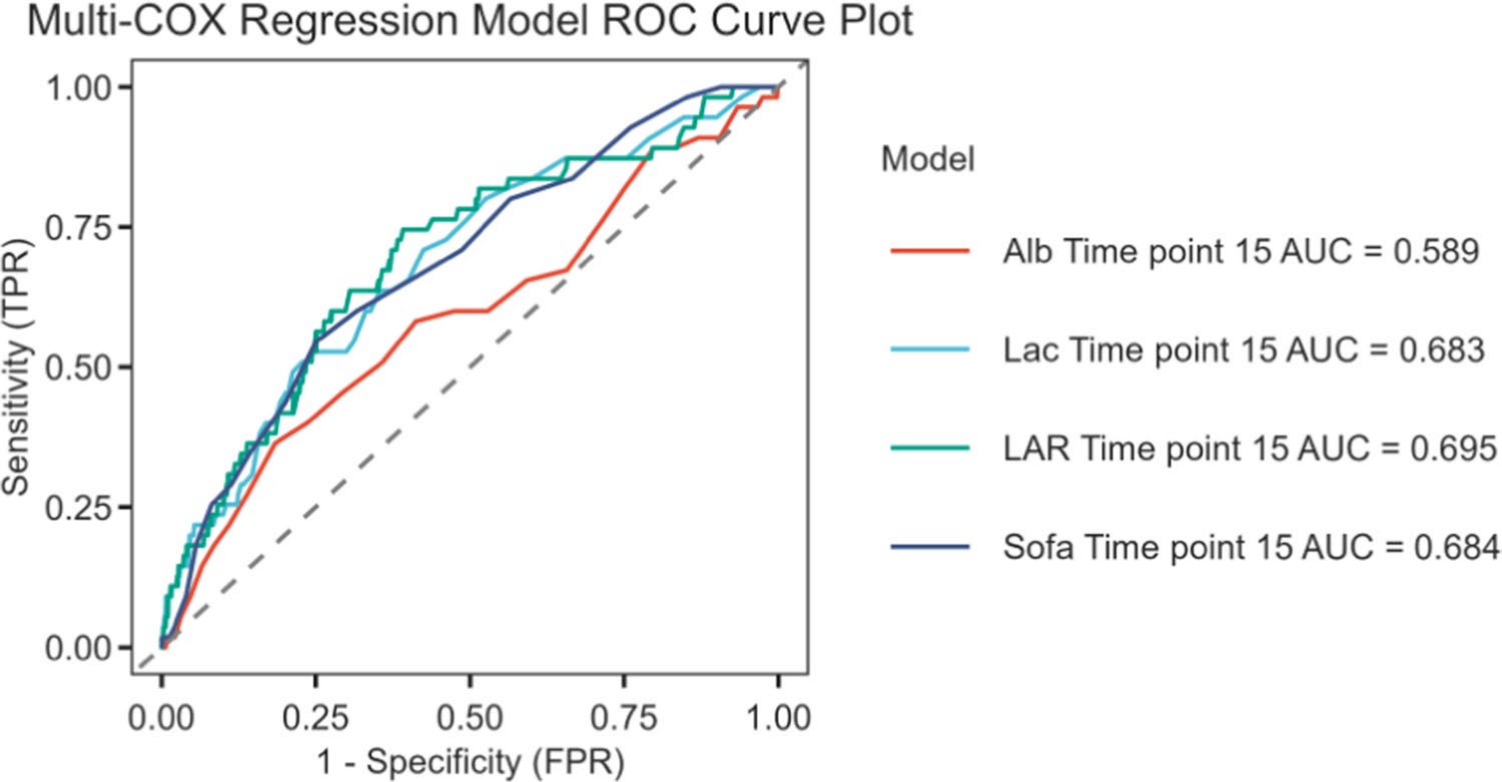
ROC curves of LAR correlate for predicting in-30day mortality. The green solid line indicates the ROC curve of the LAR. The red solid line indicates the ROC curve for Albumin. Blue indicates the ROC curve of Lactate. *LAR* Lactate/albumin ratio; *SOFA* Sequential organ failure assessment.

**Fig. 3:**
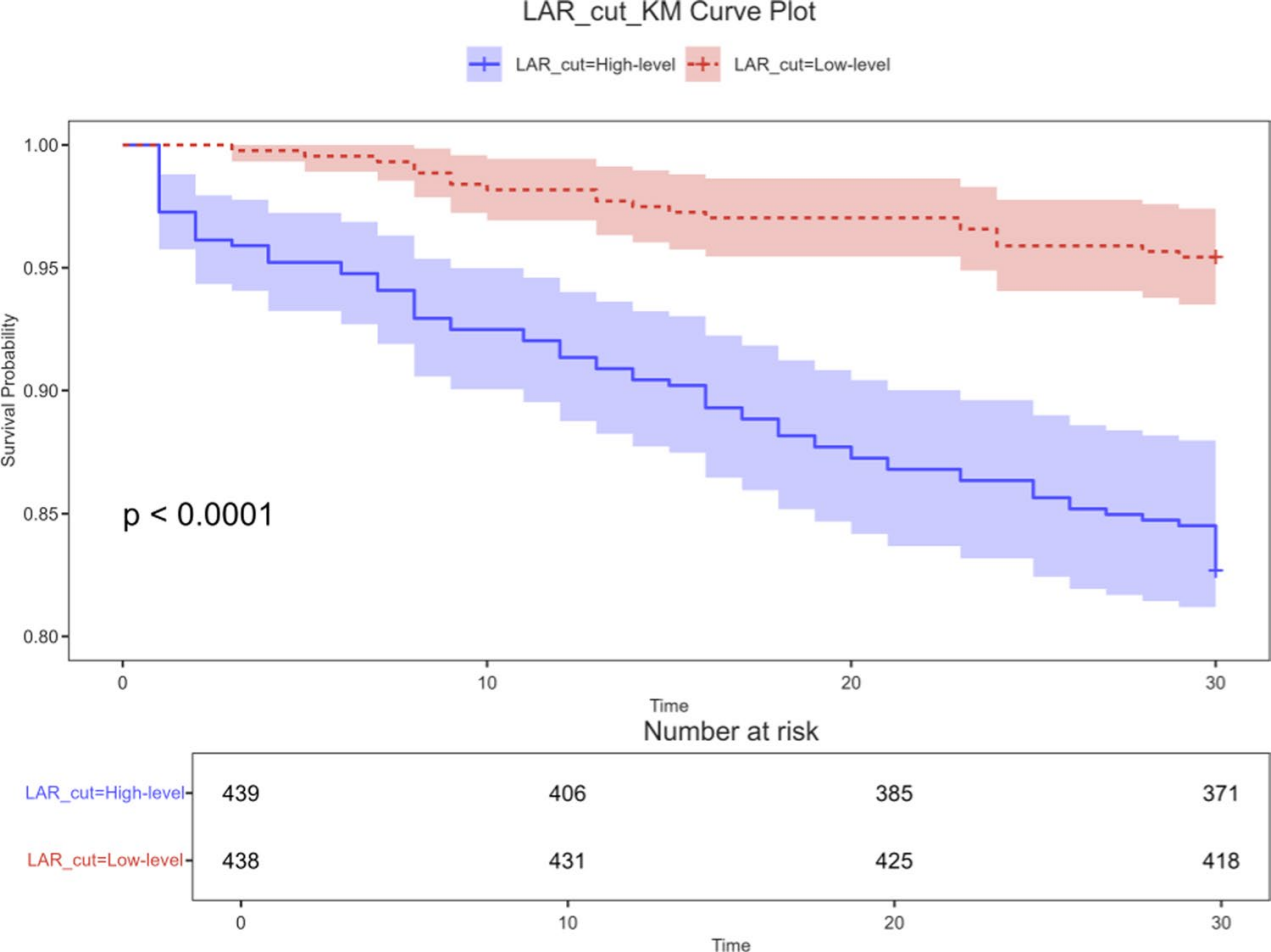
Kaplan–Meier survival analysis curves for all-cause mortality within 30-d of hospital admission.

ROC curves were generated to evaluate the predictive performance of LAR, lactate, albumin, and SOFA scores for 30-day all-cause mortality in patients with acute pancreatitis (AP). The results, shown in Fig. 2 and summarized in Table 5, indicate that LAR had a higher AUC [69.5% (95% CI 62.1–76.9%)] compared to lactate [68.3% (95% CI 60.9–75.7%)] and albumin [58.9% (95% CI 50.4–67.3%)], and was comparable to SOFA [68.4% (95% CI 61.275.5%)]. This suggests that LAR offers a notable advantage in mortality prediction. The optimal LAR cutoff was identified as 0.911, with a sensitivity of 74.5% and specificity of 60.8%. Using the median cutoff value of 0.69, patients were categorized into high LAR (≥ 0.69, n = 438) and low LAR (< 0.69, n = 439) groups. Kaplan–Meier survival curves (Fig. 3) demonstrated significantly greater mortality in the high LAR group (*P* < 0.001). These findings were corroborated by the eICU-CRD and *FAHFMU* validation cohort, which also showed higher mortality rates in patients with elevated LAR (*P* < 0.001) (Figs. S3, S4). When using in-hospital mortality as the endpoint, the AUCs were 0.645 (95% CI 0.608–0.681) for SOFA, 0.624 (95% CI 0.586–0.661) for LAR, 0.620 (95% CI 0.582–0.658) for lactate, and 0.542 (95% CI 0.503–0.582) for albumin (Fig. S6/Table S7).

**Table 5:** Information of ROC curves in Fig. 2.

Variables	AUC	95% CI	Best_Threshold	Specificity	Sensitivity
LAR	0.695	0.621–0.769	0.911	0.608	0.745
SOFA	0.684	0.612–0.755	1.475	0.749	0.545
Lactate	0.683	0.609–0.757	0.879	0.575	0.709
Albumin	0.589	0.504–0.673	1.345	0.816	0.364

Thresholds were derived from standardized predictors. *AUC* Area under the curve; *CI* Confidence interval; *ROC* Receiver operating characteristic.

### Dose–response relationships

**Fig. 4:**
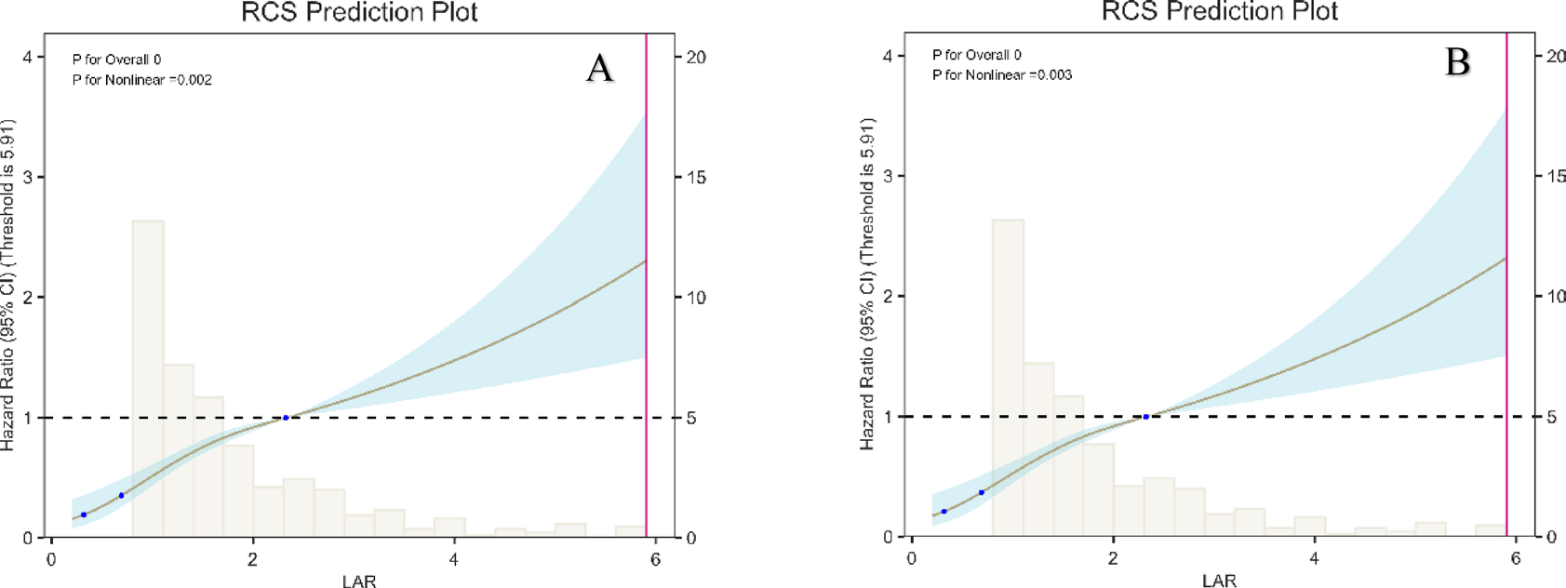
Restricted cubic spline (RCS) depicting the nonlinear association between the lactate-to-albumin ratio (LAR) and 30-day mortality in AP-AKI. (A) Unadjusted model. (B) Multivariable-adjusted model (covariates as in Table 4). The solid line shows the estimated hazard ratio and the shaded area the 95% confidence interval; the histogram displays the distribution of LAR.

We used a restricted cubic spline (RCS) model to explore the relationship between LAR and 30-day mortality in patients with AP-AKI (Fig. 4). Covariates listed in Table 4 were adjusted for in the model. A nonlinear association between LAR and 30-day mortality was observed (*P* for nonlinearity < 0.05), and a turning point was identified at a LAR value of 0.756 (Table 6). LAR was positively associated with the risk of in-hospital mortality both below and above the threshold. However, the magnitude of the association differed significantly on either side of the threshold.

**Table 6:** Threshold effect analysis of LAR on 30-day mortality in AP-AKI patients.

Threshold of LAR	HR	95% CI	*P*	Log-likelihood ratio test
< 0.765	24.687	4.475–136.202	< 0.001	< 0.001
≥ 0.765	1.304	1.178–1.443	< 0.001	

### Subgroup analysis

**Fig. 5:**
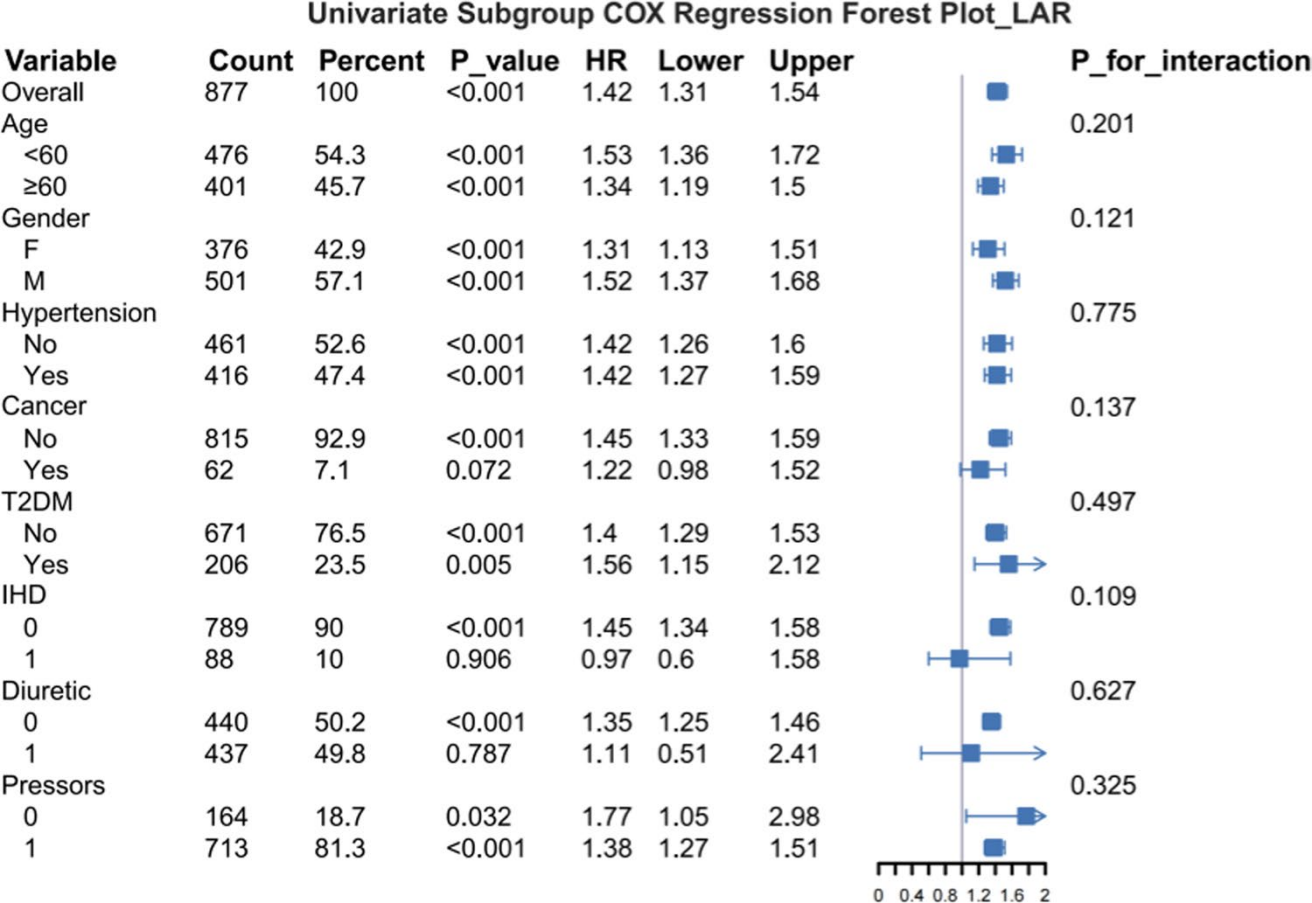
Forest plot for subgroup analysis of the relationship between hospital mortality and LAR.T2DM, Type 2 Diabetes Mellitus; IHD, Ischemic Heart Disease.

Figure 5 illustrates the stability of the association between LAR and 30-day all-cause mortality in acute pancreatitis patients across different subgroups. Stratified analyses by age, gender, hypertension, type 2 diabetes mellitus (T2DM), cancer, ischemic heart disease (IHD), diuretic use, and vasopressor administration revealed no significant interactions between LAR and these factors (P for interaction ranging from 0.109 to 0.775), supporting LAR as an independent prognostic marker. Similarly, in the eICU-CRD validation cohort, no significant interactions were detected between LAR and any predefined subgroups (Fig. S5).

## Discussion

This study examined the association between the lactate/albumin ratio (LAR) and 30-day all-cause mortality in patients with acute pancreatitis–associated acute kidney injury (AP-AKI). Our main findings indicate that higher LAR levels were significantly correlated with increased mortality, and after adjusting for potential confounders—age, sex, comorbidities, and disease severity—LAR remained an independent predictor of 30-day mortality (HR 1.172, *P* < 0.05). Receiver operating characteristic (ROC) analysis showed that LAR’s predictive accuracy (AUC 0.695) surpassed that of lactate alone (AUC 0.683), albumin alone (AUC 0.589), and the Sequential Organ Failure Assessment (SOFA) score (AUC 0.684). Subsequently, Kaplan–Meier survival curves indicated that higher LAR levels were associated with increased mortality. The RCS model further revealed a nonlinear relationship between LAR and mortality in patients with AP-AKI. These results suggest that LAR may serve as a valuable biomarker for early risk stratification in AP-AKI patients. Notably, the discriminative ability of LAR for 30-day mortality is modest (AUC ≈ 0.70), which is below commonly preferred clinical thresholds. Thus, LAR is better positioned as a simple, accessible adjunct for rapid risk flagging rather than a replacement for comprehensive scores such as SOFA.

Consistent with contemporary reviews, the higher in-hospital (late) mortality in AP-AKI likely reflects complications accruing beyond the second week—particularly infected pancreatic necrosis (IPN), which predominates after day 14 and carries substantially higher mortality (≈20–30%) than sterile necrosis—together with secondary infections, septic shock, and persistent/new organ failure during prolonged hospitalization; consequently, 30-day endpoints censor a proportion of these late events, whereas in-hospital endpoints capture them^14,15^.

Our findings are consistent with prior studies in both acute pancreatitis and sepsis-associated AKI. For instance, Liu et al. retrospectively analyzed the MIMIC-IV database and found that LAR was an independent predictor of 28-day all-cause mortality in acute pancreatitis patients, with an optimal cutoff of 1.1124 and an AUC of 74.26% (95% CI 67.02–81.50%)^10^. Although our cohort focused specifically on the AP patients who developed AKI, the similar predictive strength and cutoff value reinforce LAR’s robustness in AP-related complications.

In sepsis-associated AKI (SA-AKI), Wang et al. reported that LAR was linked to higher in-hospital, 30-day, and 90-day mortality in a nonlinear fashion, with a threshold effect at an LAR of 0.55 (HR 1.34, 95% CI 1.23–1.45, *P* < 0.001)^8^. Likewise, Zhu et al., in a multicenter eICU study, identified LAR as an independent predictor of in-hospital mortality in critically ill AKI patients (AUC 0.717; 95% CI 0.701–0.736)^16^. Another investigation further validated LAR’s prognostic value in SA-AKI, noting a threshold effect at an LAR of 2.1 (HR 1.5, 95% CI 1.2–1.8, *P* < 0.001)^17^. Together, these studies underscore LAR’s consistent prognostic significance across diverse AKI populations, despite variations in optimal cutoff values driven by different patient cohorts and study designs.

Furthermore, the application of LAR in other critical illnesses further supports its potential as a broadly applicable biomarker. For example, LAR has been shown to correlate with mortality in patients with community-acquired pneumonia^18^ and sepsis-induced myocardial injury^19^. These findings suggest that by capturing the combined effects of tissue hypoperfusion and systemic inflammation, LAR may have prognostic value across a variety of critical conditions.

The efficacy of LAR as a prognostic marker can be explained by the underlying pathophysiological mechanisms it reflects. Lactate is a marker of anaerobic metabolism that typically rises in shock, tissue hypoperfusion, or hypoxic states^20^. In AP-AKI patients, the inflammatory storm triggered by acute pancreatitis and the subsequent kidney injury may lead to systemic hypoperfusion, thereby elevating lactate levels. On the other hand, the pathogenesis of acute pancreatitis is closely linked to oxidative stress: activation of the inflammatory response and recruitment of inflammatory cells cause tissue damage^21^. Albumin, a negative acute-phase protein, is inversely correlated with the degree of inflammation and reflects both nutritional status and hepatic synthetic function^22^. It also promotes the production of anti-inflammatory mediators—such as lipoxins, resolvins, and protectins—that facilitate tissue repair, a process that consumes substantial amounts of albumin. This helps explain why hypoalbuminemia is associated with poor outcomes. Indeed, hypoalbuminemia is common in acute pancreatitis and is linked to disease severity and increased mortality^6^. By combining lactate and albumin, LAR may more comprehensively reflect the interplay between metabolic derangement and inflammatory response than either parameter alone, thereby enhancing its predictive power.

Clinically, LAR’s appeal lies in its simplicity and accessibility. Because lactate and albumin are routinely measured in clinical practice, calculating LAR requires no additional tests or resources. This makes it particularly useful for rapid risk assessment in emergency or resource-limited settings. Moreover, the predictive performance of LAR is comparable to that of the SOFA score, suggesting it could serve as a quick screening tool to identify high-risk patients and guide more intensive monitoring or therapeutic interventions—such as early aggressive fluid resuscitation, vasopressor support, or initiation of renal replacement therapy.

Compared with other biomarkers, LAR offers distinct advantages. For instance, the blood urea nitrogen/albumin ratio (BAR) has also demonstrated prognostic value in acute pancreatitis patients, with AUCs of 0.74 for 30-day mortality and 0.68 for 1-year mortality^23^. However, BAR requires additional assessment of renal function, whereas LAR depends solely on routine blood tests. Moreover, LAR’s predictive accuracy surpasses that of lactate or albumin alone, underscoring its unique ability to integrate metabolic and inflammatory information.

Nevertheless, this study has several limitations. First, despite statistical significance, the AUC of LAR is only modest, underscoring that LAR should complement rather than replace comprehensive severity scores; future studies should test combined or serial/dynamic-LAR strategies. Second, although we used multiple datasets (MIMIC-IV/III, eICU-CRD, and a single-center FAHFMU cohort), all cohorts are retrospective EHR–based and geographically constrained; the external validation samples are relatively small and, in the case of FAHFMU, single-center. These factors may limit generalizability across regions, health-care systems, and AP-AKI case mixes. Third, we examined only the first LAR measurement upon ICU admission, precluding analysis of how dynamic changes in LAR over time might affect outcomes. In addition, we did not report stage-specific ROC curves to avoid spectrum-related instability and multiplicity-driven overinterpretation. Taken together, LAR should be viewed as a complement to existing scoring systems rather than a replacement.

Future research should aim to validate these findings in prospective, multicenter cohorts with larger and more diverse patient populations. Investigations into longitudinal LAR trends and their associations with clinical outcomes are warranted, as is evaluation of whether LAR can guide therapeutic decisions or improve the management of AP-AKI patients. Additionally, studying LAR’s performance across other AKI subtypes and comparing it to emerging biomarkers—such as the C-reactive protein/albumin ratio—may further refine prognostic assessment in AP-AKI^24^, and exploring its role in longer-term outcomes (e.g., 90-day or 1-year mortality) could elucidate its broader clinical utility.

## Conclusion

In summary, this study demonstrates that LAR is a significant independent predictor of 30-day all-cause mortality in AP-AKI patients, and its simplicity and accessibility make it a powerful tool for risk assessment. Although further research is needed to validate these findings, the potential of LAR in managing this high-risk patient population should not be overlooked.

## Supplementary Information

Supplementary Information.

## Data Availability

The datasets presented in this study can be found in online repositories. The names of the repository/repositories and accession number(s) can be found in the article/supplementary material. The data involved in this study from the First Affiliated Hospital of Fujian Medical University are available from the corresponding author on reasonable request.
