# Supplementary material for: Association between lactate-to-albumin ratio and 30-day all-cause mortality in patients with acute pancreatitis-associated acute kidney injury
Source: Sci Rep. 2026 Mar 11;16:13127. doi: 10.1038/s41598-026-42882-5 (PMC13099965; doi:10.1038/s41598-026-42882-5)
Supplement: Supplementary file 1 — Supplementary Information. [file 41598_2026_42882_MOESM1_ESM.docx]

**Table S1**. Baseline data of included patients in the eICU-CRD data set.

| Variable | Total(n=127) | 30-dsurvivors(n=103) | 30-d non-survivors(n=24) | *p* |
| --- | --- | --- | --- | --- |
| Age (yr) | 55.00 (44.00 - 64.00) | 54.00 (44.00 - 63.00) | 60.50 (44.50 - 68.50) | 0.216 |
| height (cm) | 172.70 (165.10 - 180.30) | 172.70 (165.10 - 180.30) | 170.10 (160.00 - 177.80) | 0.203 |
| weight (kg) | 89.50 (74.50 - 103.80) | 90.70 (75.70 - 103.87) | 85.45 (69.45 - 96.80) | 0.208 |
| Sofa score | 7.00 (4.00 - 11.00) | 6.00 (4.00 - 10.00) | 10.50 (7.00 - 14.00) | 0.002 |
| Saps II score | 42.00 (30.00 - 52.00) | 40.00 (27.00 - 50.00) | 50.50 (43.50 - 66.00) | <0.001 |
| Hct (%) | 37.80 (31.80 - 42.30) | 37.80 (32.10 - 42.30) | 37.20 (31.05 - 42.20) | 0.642 |
| Hb (g/dL) | 12.60 (10.60 - 14.70) | 12.70 (10.70 - 14.70) | 11.80 (10.55 - 14.55) | 0.642 |
| Plt (×10^9 /L) | 270.00 (185.00 - 397.00) | 279.00 (207.00 - 406.00) | 188.50 (140.00 - 327.50) | 0.018 |
| Lac (mmol/L) | 2.60 (1.50 - 4.50) | 2.30 (1.40 - 3.60) | 4.95 (2.90 - 8.65) | <0.001 |
| LAR | 0.90 (0.50 - 1.50) | 0.81 (0.48 - 1.30) | 1.55 (1.00 - 3.45) | <0.001 |
| Alb (g/dL) | 2.90 (2.50 - 3.20) | 2.90 (2.60 - 3.30) | 2.90 (2.45 - 3.15) | 0.412 |
| INR | 1.40 (1.10 - 1.70) | 1.30 (1.10 - 1.60) | 1.70 (1.40 - 2.30) | <0.001 |
| Gender, n (p%) |  |  |  | 0.027 |
| Female | 49.00 (38.58%) | 35.00 (33.98%) | 14.00 (58.33%) |  |
| Male | 78.00 (61.42%) | 68.00 (66.02%) | 10.00 (41.67%) |  |
| IHD, n (p%) |  |  |  | 0.536 |
| No | 118.00 (92.91%) | 95.00 (92.23%) | 23.00 (95.83%) |  |
| Yes | 9.00 (7.09%) | 8.00 (7.77%) | 1.00 (4.17%) |  |
| COPD, n (p%) |  |  |  | 0.165 |
| No | 119.00 (93.70%) | 98.00 (95.15%) | 21.00 (87.50%) |  |
| Yes | 8.00 (6.30%) | 5.00 (4.85%) | 3.00 (12.50%) |  |
| Stroke, n (p%) |  |  |  | 0.257 |
| No | 125.00 (98.43%) | 102.00 (99.03%) | 23.00 (95.83%) |  |
| Yes | 2.00 (1.57%) | 1.00 (0.97%) | 1.00 (4.17%) |  |
| Cancer, n (p%) |  |  |  | 0.491 |
| No | 125.00 (98.43%) | 101.00 (98.06%) | 24.00 (100.00%) |  |
| Yes | 2.00 (1.57%) | 2.00 (1.94%) | 0.00 (0.00%) |  |
| T2DM, n (p%) |  |  |  | 0.798 |
| No | 113.00 (88.98%) | 92.00 (89.32%) | 21.00 (87.50%) |  |
| Yes | 14.00 (11.02%) | 11.00 (10.68%) | 3.00 (12.50%) |  |
| HTN, n (p%) |  |  |  | 0.019 |
| No | 99.00 (77.95%) | 76.00 (73.79%) | 23.00 (95.83%) |  |
| Yes | 28.00 (22.05%) | 27.00 (26.21%) | 1.00 (4.17%) |  |
| Ventilation, n (p%) |  |  |  | 0.016 |
| No | 80.00 (62.99%) | 70.00 (67.96%) | 10.00 (41.67%) |  |
| Yes | 47.00 (37.01%) | 33.00 (32.04%) | 14.00 (58.33%) |  |
| Antibiotic, n (p%) |  |  |  | 0.699 |
| No | 68.00 (53.54%) | 56.00 (54.37%) | 12.00 (50.00%) |  |
| Yes | 59.00 (46.46%) | 47.00 (45.63%) | 12.00 (50.00%) |  |
| Diuretic, n (p%) |  |  |  | 0.617 |
| No | 79.00 (62.20%) | 63.00 (61.17%) | 16.00 (66.67%) |  |
| Yes | 48.00 (37.80%) | 40.00 (38.83%) | 8.00 (33.33%) |  |
| Pressors, n (p%) |  |  |  | 0.544 |
| No | 86.00 (67.72%) | 71.00 (68.93%) | 15.00 (62.50%) |  |
| Yes | 41.00 (32.28%) | 32.00 (31.07%) | 9.00 (37.50%) |  |

**Table S2**. Baseline data of included patients in the *FAHFMU* data set.

| Variable | Total(n=127) | 30-dsurvivors(n=103) | 30-d non-survivors(n=24) | *p* |
| --- | --- | --- | --- | --- |
| Age (yr) | 64.00 (50.00 - 76.00) | 67.00 (50.50 - 77.00) | 60.00 (47.00 - 74.00) | 0.493 |
| Hct (%) | 0.36 (0.30 - 0.43) | 0.37 (0.32 - 0.43) | 0.30 (0.24 - 0.44) | 0.032 |
| Hb (g/L) | 118.00 (98.00 - 141.00) | 121.00 (102.50 - 141.00) | 98.00 (80.00 - 143.00) | 0.033 |
| Plt (×10^9 /L) | 189.00 (125.00 - 245.00) | 192.50 (136.50 - 247.00) | 163.00 (97.00 - 233.00) | 0.197 |
| Lac (mmol/L) | 1.40 (1.00 - 3.16) | 1.15 (0.90 - 1.95) | 4.81 (3.30 - 9.10) | <0.001 |
| LAR | 0.04 (0.03 - 0.09) | 0.03 (0.03 - 0.06) | 0.20 (0.11 - 0.30) | <0.001 |
| Alb (g/L) | 33.80 (30.40 - 38.70) | 34.90 (32.10 - 39.80) | 28.90 (27.00 - 32.30) | <0.001 |
| INR | 1.18 (1.08 - 1.31) | 1.14 (1.07 - 1.28) | 1.49 (1.24 - 2.13) | <0.001 |
| Gender, n (p%) |  |  |  | 0.609 |
| Female | 47.00 (31.54%) | 39.00 (32.50%) | 8.00 (27.59%) |  |
| Male | 102.00 (68.46%) | 81.00 (67.50%) | 21.00 (72.41%) |  |
| IHD, n (p%) |  |  |  | 0.956 |
| No | 134.00 (89.93%) | 108.00 (90.00%) | 26.00 (89.66%) |  |
| Yes | 15.00 (10.07%) | 12.00 (10.00%) | 3.00 (10.34%) |  |
| COPD, n (p%) |  |  |  | 0.540 |
| No | 146.00 (97.99%) | 118.00 (98.33%) | 28.00 (96.55%) |  |
| Yes | 3.00 (2.01%) | 2.00 (1.67%) | 1.00 (3.45%) |  |
| Stroke, n (p%) |  |  |  | 0.723 |
| No | 142.00 (95.30%) | 114.00 (95.00%) | 28.00 (96.55%) |  |
| Yes | 7.00 (4.70%) | 6.00 (5.00%) | 1.00 (3.45%) |  |
| Cancer, n (p%) |  |  |  | 0.454 |
| No | 125.00 (83.89%) | 102.00 (85.00%) | 23.00 (79.31%) |  |
| Yes | 24.00 (16.11%) | 18.00 (15.00%) | 6.00 (20.69%) |  |
| T2DM, n (p%) |  |  |  | 0.565 |
| No | 112.00 (75.17%) | 89.00 (74.17%) | 23.00 (79.31%) |  |
| Yes | 37.00 (24.83%) | 31.00 (25.83%) | 6.00 (20.69%) |  |
| HTN, n (p%) |  |  |  | 0.024 |
| No | 80.00 (53.69%) | 59.00 (49.17%) | 21.00 (72.41%) |  |
| Yes | 69.00 (46.31%) | 61.00 (50.83%) | 8.00 (27.59%) |  |
| Ventilation, n (p%) |  |  |  | <0.001 |
| No | 120.00 (80.54%) | 114.00 (95.00%) | 6.00 (20.69%) |  |
| Yes | 29.00 (19.46%) | 6.00 (5.00%) | 23.00 (79.31%) |  |
| Antibiotic, n (p%) |  |  |  | 0.018 |
| No | 20.00 (13.42%) | 20.00 (16.67%) | 0.00 (0.00%) |  |
| Yes | 129.00 (86.58%) | 100.00 (83.33%) | 29.00 (100.00%) |  |
| Diuretic, n (p%) |  |  |  | 0.179 |
| No | 68.00 (45.64%) | 58.00 (48.33%) | 10.00 (34.48%) |  |
| Yes | 81.00 (54.36%) | 62.00 (51.67%) | 19.00 (65.52%) |  |
| Pressors, n (p%) |  |  |  | <0.001 |
| No | 108.00 (72.48%) | 105.00 (87.50%) | 3.00 (10.34%) |  |
| Yes | 41.00 (27.52%) | 15.00 (12.50%) | 26.00 (89.66%) |  |

**Table S3** Multivariate Cox regression analysis was performed in the eICU-CRD validation cohort to identify risk factors for 30-day mortality.

| Variables | Hazard Ratio(HR) | 95%CI | P |
| --- | --- | --- | --- |
| Gender | 0.339 | 0.140-0.824 | 0.017 |
| HTN | 0.199 | 0.026-1.536 | 0.122 |
| LAR | 1.376 | 1.129-1.678 | 0.002 |
| Plt | 0.995 | 0.991-0.999 | 0.028 |
| Sofa score | 1.055 | 0.950-1.172 | 0.319 |
| Ventilation | 1.434 | 0.558-3.684 | 0.454 |

**Table S4** Multivariate Cox regression analysis was performed in the *FAHFMU* validation cohort to identify risk factors for 30-day mortality.

| Variables | Hazard Ratio(HR) | 95%CI | P |
| --- | --- | --- | --- |
| Alb | 0.878 | 0.817-0.943 | 0.001 |
| Hct | 0.077 | 0.001-4.361 | 0.214 |
| HTN | 0.615 | 0.255-1.483 | 0.279 |
| INR | 1.206 | 0.892-1.632 | 0.224 |
| LAR | 14.076 | 1.963-100.938 | 0.009 |


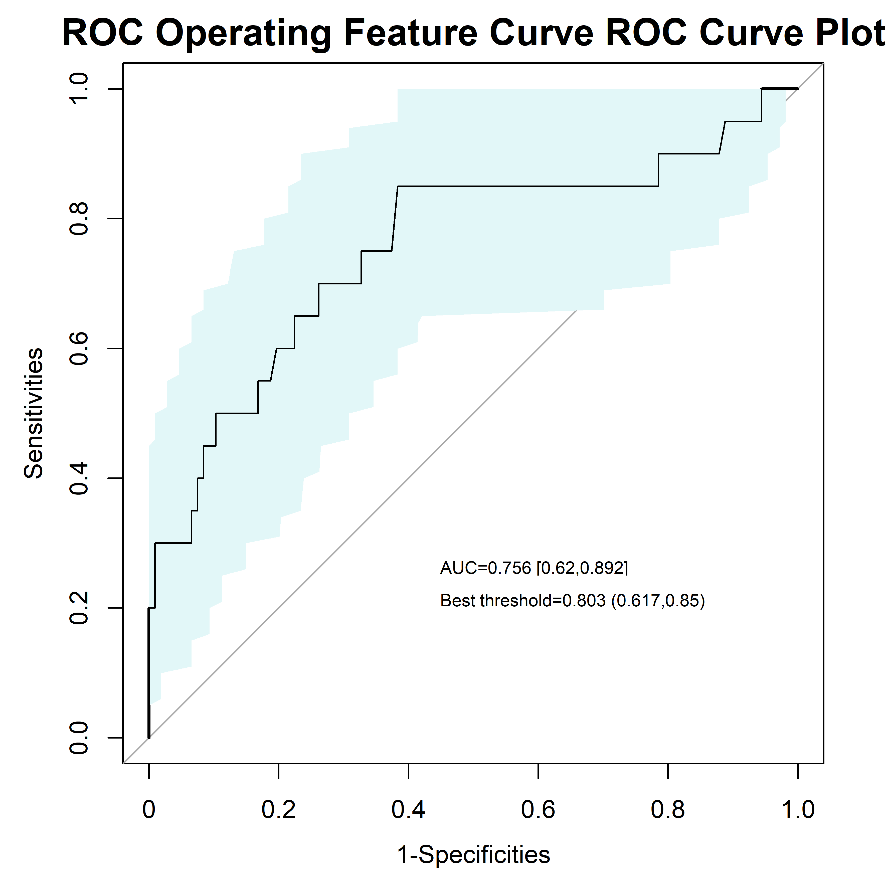


**Fig.S1** ROC curves of LAR correlate for predicting in-hospital mortality in the eICU-CRD.

| **Variables** | **AUC** | **95%CI** | **Best_Threshold** | **Specificity** | **Sensitivity** |
| --- | --- | --- | --- | --- | --- |
| LAR | 0.756 | 0.620-0.892 | 0.803 | 0.617 | 0.85 |

**TableS5** Information of ROC curves in Fig.S1.


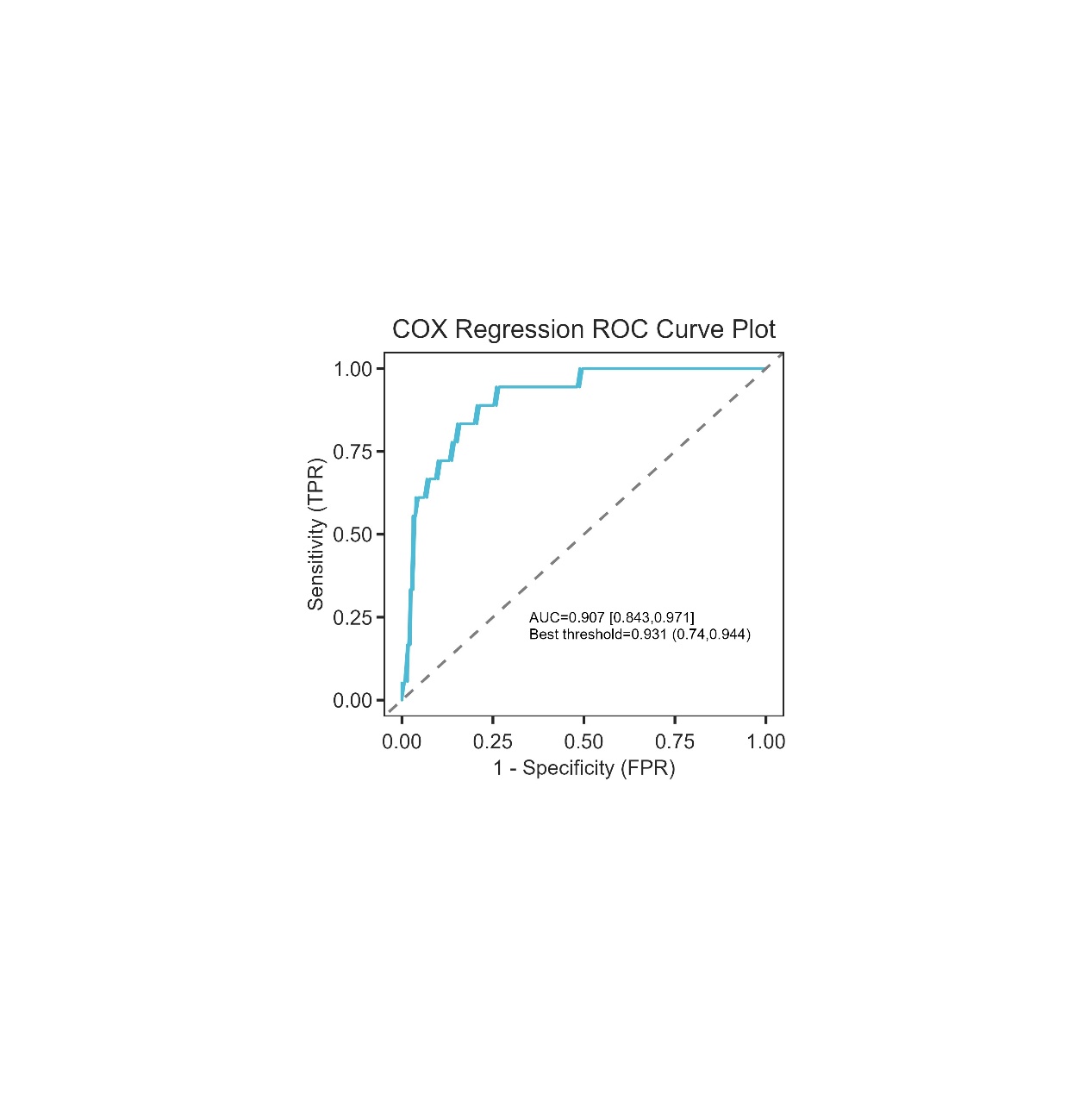


**Fig.S2** ROC curves of LAR correlate for predicting in-hospital mortality in the *FAHFMU*.

| **Variables** | **AUC** | **95%CI** | **Best_Threshold** | **Specificity** | **Sensitivity** |
| --- | --- | --- | --- | --- | --- |
| LAR | 0.907 | 0.843-0.971 | 0.930 | 0.740 | 0.944 |

**TableS6** Information of ROC curves in Fig.S2.


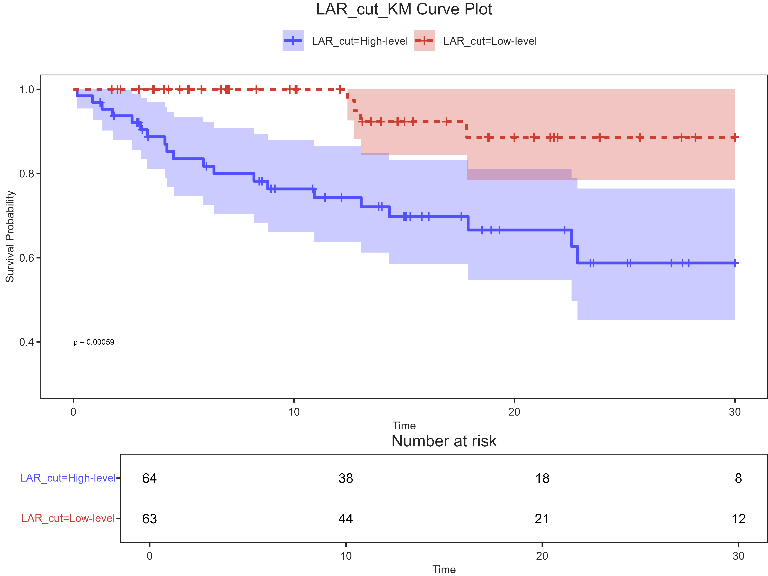


**Fig.S3** Kaplan-Meier survival analysis curves for all-cause mortality within 30-d of hospital admission in the eICU-CRD.

**Fig.S4** Kaplan-Meier survival analysis curves for all-cause mortality within 30-d of hospital admission in the *FAHFMU*.


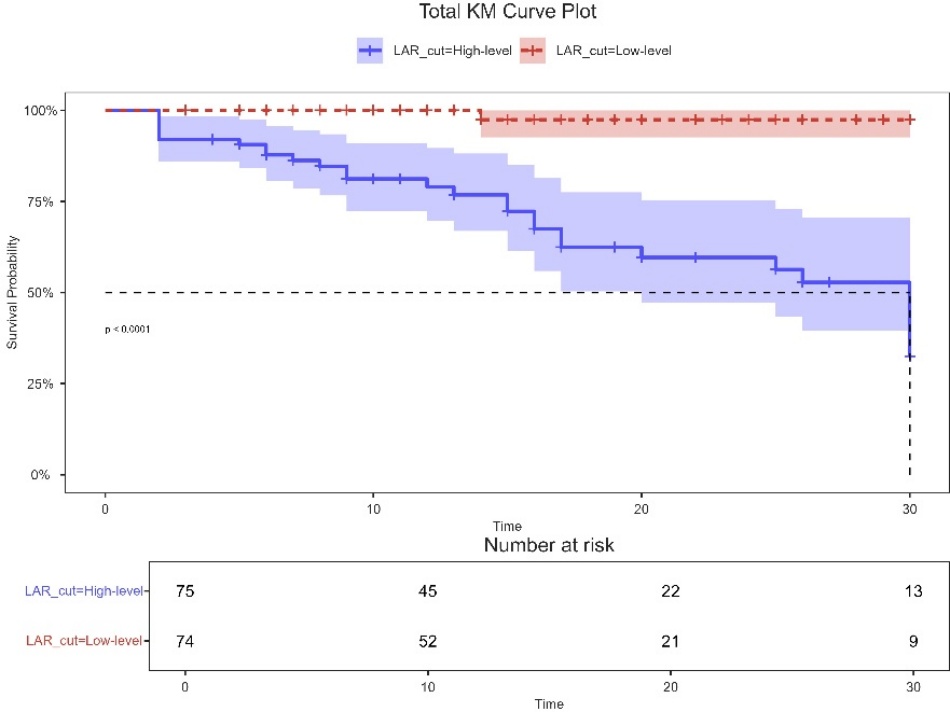


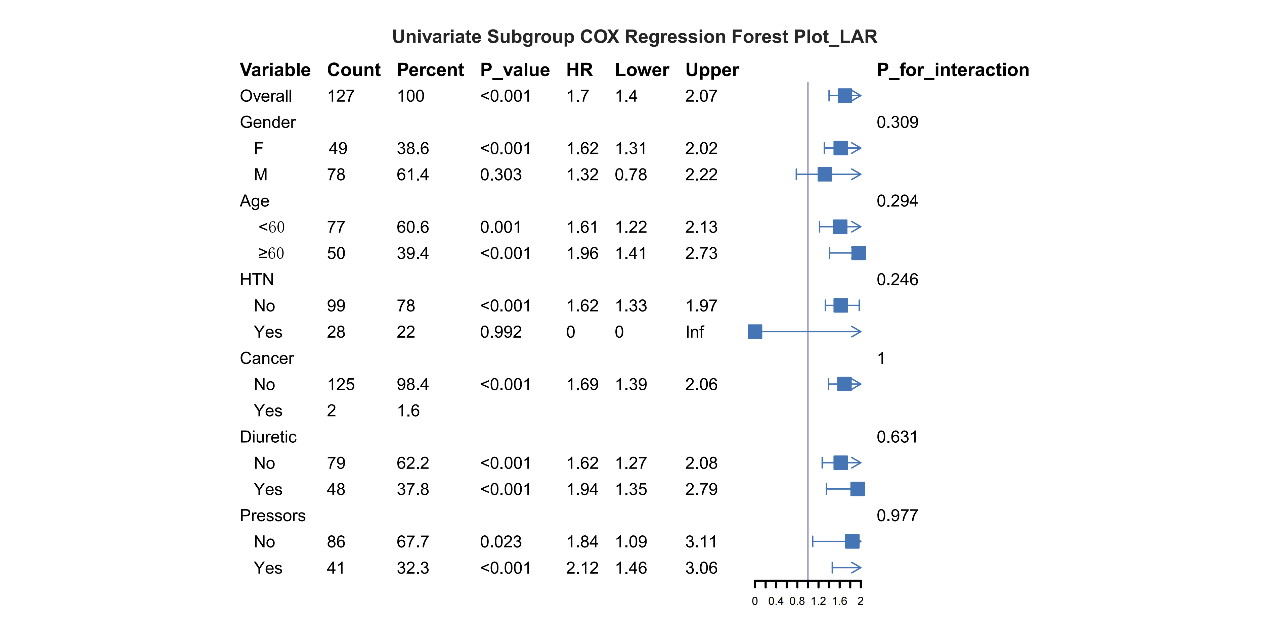


**Fig.S5** Forest plot for subgroup analysis of the relationship between hospital mortality and LAR in the eICU-CRD.

**Fig.S6** ROC curves of LAR correlate for predicting in-hospital mortality. The green solid line indicates the ROC curve of the LAR. The red solid line indicates the ROC curve for Albumin. Blue indicates the ROC curve of Lactate.


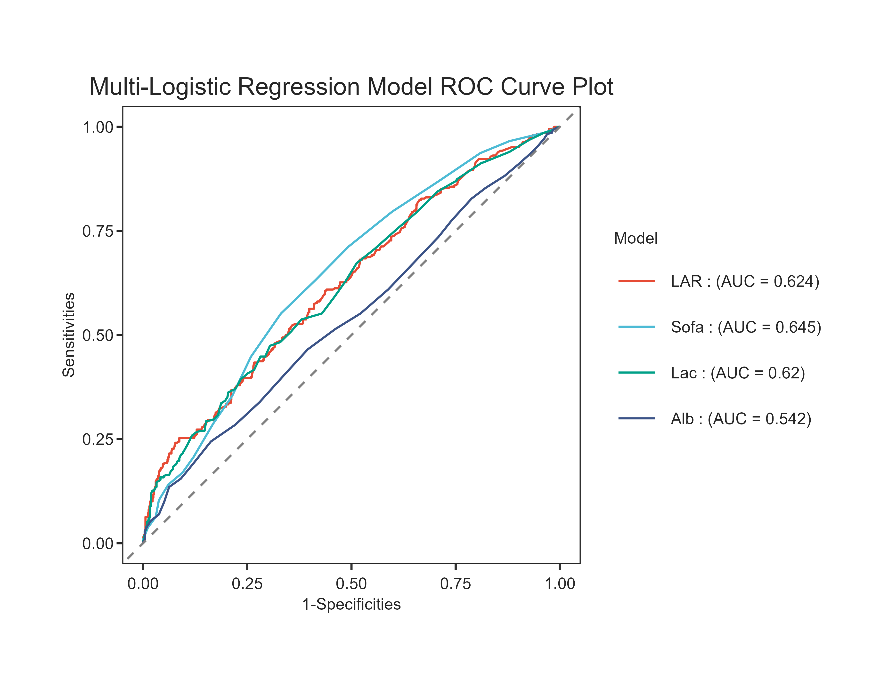


**TableS7** Information of ROC curves in Figure S6.

| **Variables** | **AUC** | **95%CI** | **Best_Threshold** | **Specificity** | **Sensitivity** |
| --- | --- | --- | --- | --- | --- |
| LAR | 0.624 | 0.586-0.661 | 0.353 | 0.563 | 0.606 |
| SOFA | 0.645 | 0.608-0.681 | 0.405 | 0.669 | 0.551 |
| Lactate | 0.620 | 0.582-0.658 | 0.377 | 0.696 | 0.474 |
| Albumin | 0.542 | 0.503-0.582 | 0.432 | 0.837 | 0.244 |
